# Early bone loss in patients with obstructive sleep apnea: a cross-sectional study

**DOI:** 10.1186/s12890-024-02848-7

**Published:** 2024-01-11

**Authors:** Yixian Qiao, Junwei Guo, Jinmei Luo, Rong Huang, Xiaona Wang, Linfan Su, Weibo Xia, Yi Xiao

**Affiliations:** 1 Department of Respiratory and Critical Care Medicine, Peking Union Medical College Hospital, Chinese Academy of Medical Sciences & Peking Union Medical College, No. 1 Shuaifuyuan, Dongcheng District, Beijing, 100730 China; 2 Department of Respiratory and Critical Care Medicine, Peking University Third Hospital, Beijing, 100191 China; 3 Department of Endocrinology, NHC Key Laboratory of Endocrinology, Peking Union Medical College Hospital, Chinese Academy of Medical Sciences & Peking Union Medical College, Beijing, 100730 China

**Keywords:** Obstructive sleep apnea, Osteoporosis, Bone metabolism, High resolution peripheral quantitative computed tomography, Bone mineral density

## Abstract

**Background:**

Obstructive sleep apnea (OSA) and osteoporosis are both prevalent diseases with shared pathophysiological mechanisms and risk factors. However, the association between the two diseases is seldom studied. This study aimed to identify the link between OSA and bone metabolism.

**Methods:**

Male participants aged 30–59-years who visited the sleep clinic were continuously recruited. Polysomnography was used to evaluate sleep and respiratory conditions. Blood samples were collected to detect metabolic, inflammatory and bone turnover indicators. High-resolution peripheral quantitative computer tomography was used to measure the non-dominant lateral radius and tibia.

**Results:**

Ninety subjects were recruited. The cortical area (Ct.Ar) of tibia of the severe OSA group was significantly higher than that of the mild and moderate OSA groups (*P* = 0.06 and *P* = 0.048). There were significant differences between the four groups in terms of total volumetric bone mineral density (vBMD) (*F* = 2.990, *P* = 0.035), meta trabecular vBMD (*F* = 3.696, *P* = 0.015), trabecular thickness (Tb.Th) (*F* = 7.060, *P* = 0.000) and cortical thickness (Ct.Th) (*F* = 4.959, *P* = 0.003). The mean values of the OSA groups were lower than control group. Hypopnea index and percentage of total sleep time with SpO_2_ < 90% were both positively correlated with alkaline phosphatase (*R* = 0.213, *P* = 0.044; *R* = 0.212, *P* = 0.045). Sleep efficiency was correlated with multiple indicators of the radius.

**Conclusions:**

In non-elderly male populations, OSA patients tended to have lower vBMD, Tb.Th and Ct.Th than non-OSA patients. The negative effect of OSA may mainly affect the osteogenesis process, and is presumed to be related to sleep-related hypoxemia and sleep efficiency.

**Supplementary Information:**

The online version contains supplementary material available at 10.1186/s12890-024-02848-7.

## Background

Obstructive sleep apnea (OSA) consists of repeated apnea and hypopnea events during sleep, characterized by repeated upper airway collapse or stenosis during sleep, causing intermittent hypoxia and sleep fragmentation at night. Clinical manifestations include snoring, repeated arousals, increased nocturia, morning headache, and daytime sleepiness. Over time, it can cause memory and cognitive decline, and even sudden death. Currently, the prevalence of the disease in individuals aged 30–69 years is approximately 936 million, and 425 million adults aged 30–69 years have moderate to severe OSA [1]. The gold standard for the diagnosis of the disease is polysomnography (PSG), while the first and commonly used treatment is continuous positive airway pressure (CPAP) therapy.

Osteoporosis is a common chronic skeletal disease related to aging. It is characterized by a reduction in bone mass and destruction of bone microstructure, causing a decrease in bone strength and an increase in bone fragility [2]. It is currently believed that factors affecting bone quality include age, genetics, nutrition, vitamin and mineral deficiencies, lifestyle, smoking history, hormone levels, and medication history [3]. Furthermore, the annual direct cost is close to 18 billion US dollars, incurring a huge burden to individuals and the national economy [4].

Only a few studies have been carried out on the relationship between OSA and bone metabolism [5–8]. Researchers have suggested that hypoxia, secondary inflammation, endothelial dysfunction, oxidative stress, sleep deprivation, and leptin resistance caused by OSA can interfere with normal bone metabolism and cause osteoporosis [9–20]. However, some researchers believe that intermittent hypoxia from OSA can stimulate the mobilization of mesenchymal stem cells and enhance the osteogenic effect in animal models [5] and that the mechanical load produced by the larger body weight of OSA patients may have a certain positive effect on bone formation [21]. Therefore, the correlation between OSA and osteoporosis remains controversial, and the specific underlying mechanisms are unclear.

Dual energy X-ray absorptiometry (DEXA) is a traditional tool that is widely used in most studies evaluating bone quality in OSA patients [9]. However, this method has the following shortcomings: First, the superimposed soft tissues on the body surface cause X-ray attenuation and beam hardening artifacts. Therefore, the application of DEXA to measure the bone density of obese patients (BMI ≥ 25 kg/m^2^) has poor accuracy [22, 23]. Second, DEXA can only detect the two-dimensional characteristics of bone, thereby without discriminating between cortical bone and trabecular bone [24, 25]. HR-pQCT is a new, non-invasive, low-radiation imaging method for systematically assessing bone quality. This technology can reconstruct the three-dimensional structure of human bones and measure volumetric bone mineral density (vBMD). Its sensitivity and specificity are significantly higher than those of traditional DEXA. It can also display bone microstructure and calculate bone mechanical performance parameters [26–28]. HR-pQCT has been fully tested for its sensitivity and accuracy [29–34]. In the past ten years, its application in clinical research has increased exponentially, helping to better understand the differences in bone microstructure caused by age, sex, and various bone metabolic diseases [35].

In this study, we compared the peripheral blood indices and HR-pQCT parameters (including bone geometry parameters, vBMD, and bone microstructure) among the OSA groups of different severities and the control group, seeking to assess the correlation between OSA and osteoporosis, and to clarify the impact of OSA on bone quality.

## Materials and methods

### Patients

**Fig. 1:**
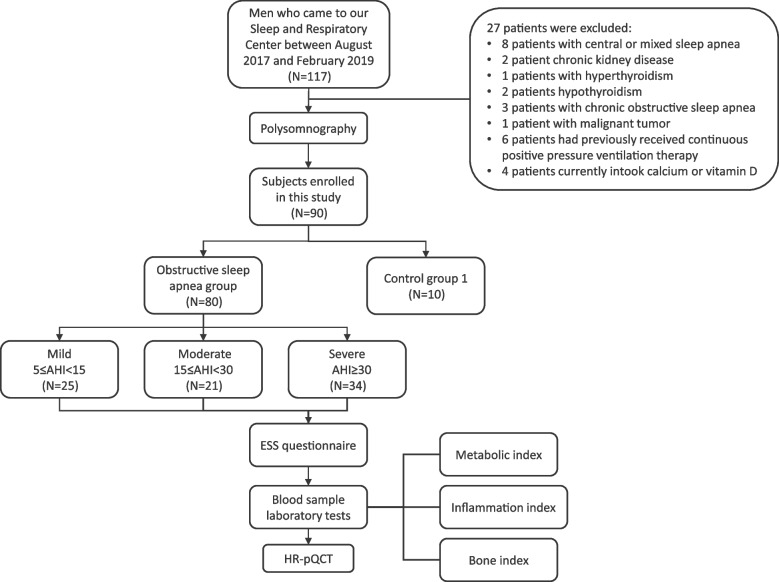
The clinical path. AHI = apnea hypopnea index; ESS = Epworth sleep score; BMI = body mass index; HR-pQCT = high-resolution peripheral quantitative computed tomography

This was a cross-sectional study. The clinical path was illustrated in Fig. 1. The study recruited male patients who came to our sleep clinic between August 2017 and February 2019, aged 30–59-years-old, and with BMI ≤ 30 kg/m^2^. Patients whose PSG results met the OSA diagnostic criteria were included in the case group, and those with normal results were in the control group. Individuals who had the following conditions were excluded: central sleep apnea; having suffered or currently suffering from diseases that affect bone metabolism; suffering from cardiovascular disease, lung disease, nervous system disease, or mental disease; having received continuous positive pressure ventilation therapy; long-term bed rest or use of wheelchairs; previous or current intake of drugs that affect bone metabolism. The study protocol was approved by the ethics committees of PUMCH (review number: ZA-1502). The whole procedure was conducted in accordance with the Declaration of Helsinki. Written informed consent was obtained from each participant in this study.

### Data collection

Baseline demographics and medical history were obtained. BMI and waist-to-hip ratio were calculated. Seated blood pressure was recorded. Patients with hypertension were defined as those with an elevated blood pressure level measured multiple times without using antihypertensive drugs or who had been diagnosed before and were taking antihypertensive drugs. Dyslipidemia patients were defined as those who had a clear history of dyslipidemia and were currently taking lipid-lowering drugs, or in whom the peripheral blood test showed abnormal blood lipid levels [36]. Patients with diabetes were defined as those who had a clear diagnosis of diabetes in the past, were currently taking hypoglycemic drugs or insulin preparations, or had a fasting blood glucose ≥ 7.0 mmol/L [37]. The Epworth sleepiness scale (ESS) was administered. Participants with ESS ≥ 9 points were considered the presence of daytime sleepiness symptoms.

To further evaluate individuals’ metabolic and inflammatory profiles, fasting blood samples were collected. Indices like fasting blood glucose (BG), total cholesterol (TC), triglycerides (TG), serum high-density lipoprotein cholesterol (HDL-C), interleukin (IL)-6, tumor necrosis factor (TNF)-α, erythrocyte sedimentation rate (ESR), and hypersensitive C-reactive protein (hs-CRP) were tested with standard procedure. Bone turnover markers including Calcium (Ca), Phosphorus (P), alkaline phosphatase (ALP), total 25- hydroxyvitamin D (T-250HD), and β-C-terminal telopeptide of type I collagen (β-CTX) were also collected.

### Polysomnography

All participants underwent nocturnal in-lab PSG (Embla N7000, USA). Sleep and respiratory events were identified according to the guidelines of the American Academy of Sleep Medicine (AASM). Apnea was defined as a decrease in respiratory airflow by 90% from baseline for more than 10 s. Hypopnea was defined as a decrease in respiratory airflow by 30% for more than 10 s, accompanied by a decrease in oxygen saturation of more than 3% or arousal. The apnea–hypopnea index (AHI) was the number of apnea or hypopnea events per hour, and AHI ≥ 5 times/hour was considered reflective of OSA. Patients with OSA were divided into mild (5 times/h ≤ AHI < 15 times/h), moderate (15 times/h ≤ AHI < 30 times/h), and severe (AHI ≥ 30 times/h) groups. This study also included the average blood oxygen saturation, lowest blood oxygen saturation, and percentage of total sleep time (TST) with SpO_2_ < 90% (%TST- SpO_2_ < 90%) as the oxygenation indicators. Sleep efficiency was defined as TST/total recording time × 100%.

### HR-pQCT

HR-pQCT (Xtreme CTII; Scanco Medical AG, Bruttisellen, Switzerland) uses the standard mode (68kVp, 1462 μA, 100 ms) with a resolution of 61 μm. The measurement sites were the tibia and radius on the non-dominant side. The forearm and calf were fixed in carbon fiber castings in the scanner platform. A total of 168 CT slices were collected for each part. The analysis and reconstruction followed the standard protocol provided by the manufacturer. Doctors were responsible for the semi-automatic contour restoration of each CT slice at every cross-sectional level to identify the periosteum boundary, and segmented the cortical bone and trabecular compartment. The following indicators were obtained: bone geometric parameters, including total area (Tt.Ar), cortical perimeter (Ct.Pm), cortical area (Ct.Ar), trabecular area (Tb.Ar). BMD parameters, including total vBMD, trabecular vBMD, cortical vBMD, external trabecular vBMD, and internal trabecular vBMD. Bone microstructure parameters, including trabecular bone volume fraction (BV/TV), trabecular number (Tb.N), trabecular thickness (Tb.Th), trabecular separation (Tb.Sp), trabecular bone heterogeneity (St. Dev of 1/Tb.N: Inhomogeneity of network, Tb.1/N.SD), cortical thickness (Ct.Th), and intra-cortical porosity (Ct.Po), and cortical pore diameter (Ct.Po.Dm) [26].

### Statistical analysis

SPSS software (v25.0, IBM, USA) was used for data analysis. Normally distributed data, skewed data, and categorical data were expressed as mean ± standard deviation, median (interquartile variance), and proportion (percentage), respectively. Comparison of means between the four groups: One-way analysis of variance was used for continuous variables with normal distribution (post-hoc comparison: Bonferroni method for those with uniform variance, and Games-Howell method for those with uneven variance). The Kruskal–Wallis H (K) test was used for continuous variables with non-normal distribution, and the chi-square test was used for categorical variables. Correlation between two variables: a Pearson correlation analysis was used for continuous variables with normal distribution; otherwise, a Spearman correlation analysis was used. A stepwise multiple regression analysis was used (introduced when *P* < 0.05, and related variables were eliminated when *P* ≥ 0.1) to identify factors that can affect bone parameters. A two-sided *P* < 0.05 was considered statistically significant.

## Results

### General clinical data and sleep-related data

A total of 90 subjects were enrolled in this study (Table 1). The sample included individuals across the range of OSA severity. The average age was 47.13 ± 5.85 years old, with no significant difference between the groups. The average BMI was 25.66 ± 2.09. kg/m^2^ with significant differences (*F* = 4.715, *P* = 0.004). Pairwise comparison (Table 2) showed the BMI of the severe OSA group was significantly higher than non-OSA (*P* = 0.036) and mild OSA group (*P* = 0.021). The proportions of OSA subjects combined with dyslipidemia were significantly higher than non-OSA subjects (*χ*^*2*^ = 10.033, *P* = 0.018). Differences of smoking and drinking between groups was insignificant.

**Table 1:** General clinical data and polysomnographic parameters of OSA and control group subjects

Items	Total	Control group	Mild OSA group	Moderate OSA group	Severe OSA group	*F/χ*^*2*^-value	*P*-value
Number	90	10	25	21	34		
Age, year	47.13 ± 5.85	45.60 ± 5.99	47.04 ± 4.61	49.05 ± 5.03	46.47 ± 6.95	1.131	0.341
BMI, kg/m^2^	25.66 ± 2.09	24.64 ± 2.33	25.09 ± 2.11	25.25 ± 2.14	26.64 ± 1.63	4.715	0.004
SBP, mmHg	125.39 ± 13.73	120.90 ± 16.27	125.56 ± 14.39	121.81 ± 16.28	128.79 ± 9.94	1.560	0.205
DBP, mmHg	85.00(80.00–95.00)	84.50(73.75–97.00)	90.00(85.00–94.50)	80.00(75.00–90.00)	85.00(80.00–95.00)	4.490	0.213
MBP, mmHg	98.15(91.90–105.00)	95.65(82.67–106.67)	102.00(95.00–106.00)	92.00(87.00–92.00)	98.65(94.58–98.65)	4.871	0.182
NC, cm	39.00(38.00–40.70)	37.50(36.00–39.25)	39.00(37.50–40.00)	40.00(38.00–41.00)	39.50(38.00–41.00)	7.401	0.060
WC, cm	91.96 ± 6.09	89.80 ± 9.94	91.04 ± 4.64	90.52 ± 6.04	94.15 ± 5.17	2.597	0.058
HC, cm	98.98 ± 5.18	99.10 ± 8.14	96.96 ± 5.80	98.57 ± 4.12	100.68 ± 3.67	2.666	0.053
WC/HC	0.93(0.90–0.96)	0.89(0.87–0.93)	0.93(0.91–0.98)	0.92(0.90–0.96)	0.94(0.91–0.96)	6.582	0.086
Hypertension, %	38.89	30.00	44.00	23.81	47.06	3.571	0.312
Diabetes, %	6.67	0.00	4.00	4.76	11.76	2.543	0.468
Dyslipidemia, %	53.33	10.00	60.00	47.62	64.71	10.033	0.018
Smoking history, %	27.78	20.00	28.00	28.57	29.41	0.354	0.950
drinking history, %	48.89	30.00	48.00	52.38	52.94	1.762	0.623
ESS	9.20 ± 4.96	8.10 ± 5.38	8.56 ± 5.10	9.14 ± 4.73	10.03 ± 5.0	0.612	0.609
AHI, /h	22.05(8.75–40.98)	1.75(0.33–3.75)	8.8(6.55–11.75)	22.3(17.75–26.1)	48.50(38.88–58.60)	81.053	0.000
AI, /h	8.50(2.78–27.28)	0.40(0.08–1.10)	3.20(1.85–5.90)	8.90(5.45–17.85)	35.85(24.38–45.18)	67.482	0.000
HI, /h	6.80(2.88–15.25)	1.00(0.00–3.15)	4.80(2.85–6.80)	12.80(4.60–15.65)	14.70(3.50–18.63)	27.987	0.000
Lowest SpO_2_, %	86.00(79.75–90.00)	92.50(86.75–96.25)	90.00(86.00–91.50)	87.00(83.00–90.00)	77.50(74.75–84.25)	44.349	0.000
Average SpO_2_, %	96.95(94.68–98.00)	97.80(96.93–98.43)	97.80(96.75–98.25)	97.00(94.50–98.15)	94.85(93.38–96.93)	24.652	0.000
TST, min	398.30(374.58–411.08)	390.35(327.58–410.55)	397.8(374.80–409.55)	393(371.70–404.85)	402.70(374.50–416.87)	2.867	0.413
% TST- SpO_2_ < 90%, %	0.30(0.00–5.50)	0.00(0.00–0.13)	0.00(0.00–0.20)	0.30(0.00–1.85)	6.85(0.90–12.88)	47.698	0.000
N1, %	14.40(8.90–22.25)	9.30(7.07–20.1)	15.70(10.30–21.35)	15.1(8.15–22.80)	13.70(9.28–23.03)	1.515	0.679
N2, %	43.53 ± 11.48	40.81 ± 16.02	41.14 ± 8.46	44.70 ± 13.48	45.37 ± 10.61	0.910	0.440
N3, %	23.12 ± 9.42	27.57 ± 10.29	25.16 ± 7.24	22.05 ± 10.62	20.97 ± 9.44	1.870	0.141
REM, %	17.11 ± 6.09	18.66 ± 5.08	17.81 ± 5.97	16.37 ± 5.91	16.61 ± 6.64	0.500	0.683
Sleep efficiency, %	92.91(87.85–95.86)	91.76(77.80–94.80)	92.89(88.73–95.55)	94.05(87.97–96.01)	93.36(88.73–96.87)	1.698	0.637

*OSA* obstructive sleep apnea, *BMI* body mass index, *SBP* systolic blood pressure, *DBP* diastolic blood pressure, *MBP* mean blood pressure, *NC* neck circumference, *WC* waist circumference, *HC* hip circumference, *ESS* Epworth Sleep Scale, *AHI* apnea–hypopnea index, *AI* apnea index, *HI* hypopnea index, *SpO*_2_ oxygen saturation, *TST* total sleep time, *% TST- SpO*_*2*_ < *90%* percentage of TST with SpO_2_ < 90%, *REM* rapid eye movement. 1 mmHg = 0.133kpa

**Table 2:** Blood test results of OSA and control group subjects

Items	Total	Control group	Mild OSA	Moderate OSA group	Severe OSA group	*F*/*χ*^*2*^-value	*P*-value
TC	4.73(4.20–5.34)	4.17(3.92–5.22)	4.65(4.16–5.32)	5.07(4.39–5.64)	4.63(4.15–5.41)	1.743	0.627
TG	1.63(1.25–2.06)	0.93(0.86–1.48)	1.83(1.34–2.39)	1.67(1.32–2.17)	1.71(1.32–2.36)	10.158	0.017
HDL-C	1.03(0.92–1.20)	1.13(1.02–1.20)	1.01(0.93–1.26)	1.00(0.95–1.16)	1.02(0.88–1.20)	1.822	0.610
BG	5.10(4.80–5.60)	5.20(4.65–5.43)	4.80(4.40–5.25)	5.10(4.80–5.65)	5.40(4.98–5.83)	10.864	0.012
IL-6	2.00(2.00–2.00)	2.00(2.00–2.00)	2.00(2.00–2.10)	2.00(2.00–2.00)	2.00(2.00–2.00)	1.221	0.748
TNF-α	7.90(6.50–10.55)	6.75(6.18–11.28)	8.10(6.20–16.35)	8.10(6.85–9.35)	7.90(6.88–10.70)	1.072	0.784
ESR	4.00(2.00–6.00)	3.00(2.75–4.50)	2.00(1.00–6.00)	4.00(2.00–5.00)	4.50(2.00–6.00)	2.228	0.526
HsCRP	1.00(0.62–1.70)	1.50(0.69–2.38)	0.98(0.53–1.62)	0.89(0.52–1.49)	1.04(0.68–2.23)	2.912	0.405
Ca	2.32 ± 0.10	2.33 ± 0.09	2.31 ± 0.09	2.27 ± 0.09	2.33 ± 0.10	2.153	0.100
P	1.16(1.05–1.24)	1.19(1.10–1.23)	1.16(1.05–1.26)	1.16(1.01–1.26)	1.16(1.05–1.22)	0.176	0.981
T-25OHD	18.10(14.70–23.05)	19.60(16.30–21.93)	16.30(15.00–24.80)	17.90(13.00–23.25)	18.90(14.68–22.55)	0.968	0.809
β-CTX	0.53 ± 0.19	0.52 ± 0.17	0.49 ± 0.14	0.54 ± 0.24	0.55 ± 0.21	0.456	0.714
ALP	79.18 ± 20.83	77.50 ± 22.56	74.56 ± 24.25	80.71 ± 20.74	82.12 ± 17.76	0.688	0.562

*OSA* obstructive sleep apnea, *TC* cholesterol, *TG* triglycerides, *HDL-C* high-density lipoprotein cholesterol, *BG* blood glucose, *IL-6* interleukin-6, *TNF-α* tumor necrosis factor- α, *ESR* erythrocyte sedimentation rate, *HsCRP* high-sensitivity C-reactive protein, *Ca* calcium, *P* blood phosphorus, *T-25OHD* total 25-hydroxyvitamin D, *β-CTX* β-I collagen carboxy-terminal peptide, *ALP* alkaline phosphatase

In terms of sleep conditions (Table 1), the average ESS score of the four groups was 9.20 ± 4.96, with no significant difference between them (*P* = 0.609). Significant differences related to the severity of OSA were found, including AHI, apnea index (AI), hypopnea index (HI), lowest blood oxygen saturation, average blood oxygen saturation, and %TST-SpO_2_ < 90%. Further pairwise analysis (Table S1) found that the lowest and average oxygen saturation in patients with severe OSA was significantly lower than those in the non-OSA and mild OSA group. % TST- SpO_2_ < 90% in the severe OSA group was also significantly higher than the other three groups. There was no significant difference regarding TST and sleep efficiency across groups.

### Blood test results

There were significant differences in TG (*χ*^*2*^ = 10.158, *P* = 0.017) and BG (*χ*^*2*^ = 10.864, *P* = 0.012) between the four groups (Table 2). Pairwise comparison (Table S2) showed that the TG of patients with severe OSA was significantly higher than the control group (*P* = 0.009), and BG was significantly higher than the mild OSA group (*P* = 0.006). There was no significant difference between the groups on inflammation and bone turnover indicators.

### HR-pQCT parameters

There were no significant differences of parameters on distal radius (Table S3). In terms of the geometric parameters of the tibia (Table 3), the four groups of patients had significant differences in Ct.Ar (*F* = 4.797, *P* = 0.04). Pairwise comparison showed that (Table S4) the Ct.Ar of the severe OSA group was significantly higher than the mild (*P* = 0.06) and moderate OSA groups (*P* = 0.048). In terms of vBMD, the four groups of patients had statistical differences in Tt.vBMD (*F* = 2.990, *P* = 0.035) and Tb.Meta.vBMD (*F* = 3.696, *P* = 0.015). OSA patients had lower values than healthy controls. Pairwise comparison showed that only Tb.Meta.vBMD in the mild OSA group was lower than non-OSA group (*P* = 0.025). In terms of bone microstructure, the four groups of patients had significant differences in Tb.Th (*F* = 7.060, *P* = 0.000) and Ct.Th (*F* = 4.959, *P* = 0.003). The mean values ​​of the three severe OSA groups were lower than non-OSA group. Pairwise comparison showed that the Tb.Th of patients with mild (*P* = 0.001) and severe OSA (*P* = 0.001) were lower than non-OSA group. The Ct.Th of the mild OSA group was significantly lower than non-OSA and severe OSA groups (*P* = 0.049 and 0.026).

**Table 3:** HR-pQCT parameters of tibial of OSA and control group subjects

Items		Total	Control group	mild OSA group	Moderate OSA group	Severe OSA group	*F/χ*^*2*^-value	*P*-value
Geometric	Tt.Ar(mm^2^)	80.27 ± 107.39	763.66 ± 93.98	829.96 ± 125.13	841.41 ± 113.65	824.10 ± 90.60	1.221	0.307
Parameters	Ct.Pm(mm)	111.88 ± 7.62	108.17 ± 7.46	111.48 ± 8.66	113.28 ± 7.84	112.49 ± 6.63	1.105	0.352
	Ct.Ar(mm^2^)	147.75 ± 22.60	153.03 ± 27.57	138.22 ± 16.83	141.20 ± 18.07	157.25 ± 23.86	4.797	0.004
	Tb.Ar(mm^2^)	678.38 ± 108.33	616.24 ± 84.37	687.58 ± 123.61	706.14 ± 122.07	672.74 ± 88.35	1.685	0.176
BMD	Tt.vBMD(mg HA/ccm)	291.13 ± 49.78	316.37 ± 48.03	274.78 ± 52.86	279.28 ± 46.77	303.04 ± 45.44	2.990	0.035
	Tb.vBMD(mg HA/ccm)	156.41 ± 36.71	173.79 ± 35.44	145.54 ± 37.98	153.34 ± 31.28	161.17 ± 37.94	1.761	0.161
	Tb.Meta.vBMD(mg HA/ccm)	223.98 ± 40.97	249.76 ± 37.75	206.54 ± 42.09	219.10 ± 30.47	232.24 ± 42.08	3.696	0.015
	Tb.Innn.vBMD(mg HA/ccm)	101.80(84.95–137.85)	125.40(88.70–135.65)	97.40(84.30–108.90)	98.40(85.60–138.00)	111.30(78.80–143.35) 2.660		0.447
	Ct.vBMD(mg HA/ccm)	905.73 ± 50.50	894.55 ± 27.19	912.27 ± 57.06	896.25 ± 56.71	910.06 ± 47.02	0.625	0.601
Microstructure	BV/TV	0.24 ± 0.05	0.26 ± 0.05	0.23 ± 0.05	0.24 ± 0.04	0.25 ± 0.05	1.723	0.168
Parameters	Tb.N(/mm)	1.26(1.14–1.37)	1.27(1.11–1.42)	1.23(1.11–1.36)	1.29(1.21–1.38)	1.27(1.14–1.38)	1.406	0.704
	Tb.Th(mm)	0.26 ± 0.02	0.28 ± 0.03	0.25 ± 0.02	0.25 ± 0.02	0.26 ± 0.02	7.060	0.000
	Tb.Sp(mm)	0.78(0.71–0.86)	0.78(0.69–0.88)	0.80(0.71–0.88)	0.76(0.72–0.82)	0.76(0.69–0.84)	1.287	0.732
	Tb.1/N.SD(mm)	0.33(0.29–0.36)	0.33(0.28–0.37)	0.33(0.29–0.37)	0.32(0.29–0.36)	0.33(0.30–0.36)	0.518	0.915
	Ct.Th(mm)	1.55 ± 0.27	1.70 ± 0.29	1.44 ± 0.21	1.46 ± 0.24	1.64 ± 0.27	4.959	0.003
	Ct.Po	0.03(0.02–0.25)	0.03(0.02–0.04)	0.02(0.01–0.03)	0.03(0.02–0.04)	0.03(0.02–0.04)	5.519	0.138
	Ct.Po.Dm(mm)	0.23(0.21–0.25)	0.24(0.23–0.27)	0.23(0.21–0.25)	0.22(0.21–0.24)	0.24(0.21–0.27)	7.263	0.064

*HR-pQCT* high-resolution peripheral quantitative computed tomography, *OSA* obstructive sleep apnea, *Tt.Ar* total area, *Ct.Pm* cortical perimeter, *Ct.Ar* cortical area, *Tb.Ar* trabecular area, *BMD* bone mineral density, *Tt.vBMD* total volumetric vBMD, *Tb.vBMD* trabecular vBMD, *Tb.Meta.vBMD* external trabecular vBMD, *Tb.Inn.vBMD* internal trabecular vBMD, *Ct.vBMD* cortical vBMD, *BV/TV* trabecular bone volume fraction, *Tb.N* trabecular number, *Tb.Th* trabecular thickness, *Tb.Sp* trabecular separation, *Tb.1/N.SD* trabecular bone heterogeneity, *Ct.Th* cortical thickness, *Ct.Po* intra-cortical porosity, *Ct.Po.Dm* cortical pore diameter

### Correlation analysis and multiple regression analysis

Correlations of general information, sleep indicators, blood test, and geometric parameters were shown in table S5 to table S9. HI and %TST-SpO_2_ < 90% were positively correlated with ALP. For the radius (Table S6, S8), age were negatively correlated with multiple radius indicators of BMD and microstructure, such as Tt.vBMD, Tb.vBMD, Ct.vBMD, and Tb.Th. BMI was positively correlated with multiple indicators, such as Ct.Ar, Tt.vBMD, Tb.vBMD, BV/TV, Tb.N, Tb.Th, and Ct.Th, but negatively correlated with Tb.Sp (*R* = -0.261, *P* = 0.013). Neck circumference was positively correlated with multiple radius geometric indices. Hip circumference was correlated with multiple radius vBMD and bone microstructure parameters. Among the sleep indicators, only AHI was positively correlated with Tb.Th (*R* = 0.210, *P* = 0.047). Multiple parameters of radius, such as Ct.Ar, Th.Ar, Tt.vBMD, and Tb.vBMD, showed a correlation with sleep efficiency. In terms of bone turnover indicators, Ca was negatively correlated with Tb.N (*R* = -0.242, *P* = 0.022). β-CTX was negatively correlated with multiple radius parameters, including Tt.vBMD, Tb.vBMD, Tb.Meta.vBMD, Tb.Inn.vBMD, and BV/TV.

For the tibia (Table S7, S9), the correlation analysis showed that age was negatively correlated with multiple indicators, such as Ct.Ar, Tt.vBMD, Tb.vBMD, BV/TV, and TB.Th. BMI was positively correlated with indicators like Ct.Ar, Tt.vBMD, Tb.vBMD, BV/TV, TB.Th, and Ct.Th. Multiple tibia parameters were positively correlated with neck circumference, hip circumference and waist-to-hip ratio. Among the indicators reflecting the severity of OSA, only AHI (*R* = 0.261, *P* = 0.013) and AI (*R* = 0.223, *P* = 0.034) showed a positive correlation with Ct.Ar. Ca was positively correlated with Tb.Meta.vBMD and Tb.Th. P was negatively correlated with Ct.vBMD. β-CTX was correlated with a number of indicators, including Tt.vBMD, Tb.vBMD, Tb.Inn.vBMD, Ct.vBMD, and Tb.1/N.SD.

Regression analysis was performed on the bone turnover indicators and HR-pQCT indicators (Tables 4 and 5). None of the relevant indicators reflecting the severity of OSA showed correlation with the indicators of HR-pQCT. Among the various indicators of HR-pQCT, most had a linear correlation with age, body type indicators (such as neck circumference and BMI), and comorbid indicators (such as diabetes history, blood lipid indicators, etc.). Sleep efficiency was correlated with multiple indices of radius, including Ct.Ar, Tt.vBMD, Tb.vBMD, BV/TV, and Ct.Th.

**Table 4:** Regression analysis of blood and bone turnover indicators

Items		*β*-value	*p*-value	*R*^*2*^-value	*F*-value	*P*-value
Ca	TC	0.326	0.002	0.106	10.453	0.002
T-25OHD^a^	Age	0.222	0.032	0.101	4.865	0.010
	HDL-C	0.215	0.038			
β-CTX	DBP	-0.249	0.018	0.062	5.807	0.018
ALP	TC	0.303	0.004	0.105	6.224	0.003
	Average SpO_2_	-0.226	0.028			

*Ca* blood calcium, *TC* total cholesterol, *T-25OHD* total 25-hydroxyvitamin D, *HDL-C* high-density lipoprotein cholesterol, *β-CTX* β-I collagen carboxy terminal peptide, *ALP* alkaline phosphatase, *DBP* diastolic blood pressure

^a^The dependent variable undergoes normal transformation (lg)

**Table 5:** Stepwise multiple regression analysis of HR-pQCT parameters

Items		*β-value*	*p*-value	*R*^*2*^-value	*F*-value	*P*-value	Items		*β*-value	*p*-value	*R*^*2*^-value	*F*-value	*P*-value
Tt.Ar	NC	0.264	0.012	0.069	6.569	0.012	Tt.Ar	NC	0.295	0.005	0.087	8.383	0.005
Ct.Ar	BMI	0.363	0	0.259	9.995	0	Ct.Pm	NC	0.296	0.005	0.088	8.45	0.005
	Sleep efficiency	0.261	0.007				Ct.Ar	BMI	0.728	0	0.381	14.691	0
	P	-0.228	0.017					NC	-0.388	0.001			
Th.Ar	NC	0.25	0.017	0.097	4.674	0.012		TG	0.218	0.014			
	Age	0.218	0.036					Age	-0.176	0.045			
Tt.vBMD	Age	-0.255	0.009	0.239	6.688	0	Tb.Ar	NC	0.289	0.005	0.12	5.945	0.004
	HDL-C	-0.187	0.056					Age	0.229	0.026			
	Sleep efficiency	0.249	0.012				Tt.vBMD	Age	-0.364	0	0.386	13.341	0
	β-CTX	-0.245	0.014					BMI	0.619	0			
Tb.vBMD	BMI	0.178	0.066	0.295	7.039	0		NC	-0.446	0			
	Age	-0.247	0.009					WC/HC	-0.219	0.015			
	Diabetes	0.22	0.022				Tb.vBMD	Age	-0.252	0.01	0.198	8.306	0
	Sleep efficiency	0.258	0.008					WC/HC	-0.312	0.002			
	β-CTX	-0.24	0.013					BMI	0.3	0.003			
Ct.vBMD	Age	-0.243	0.021	0.059	5.539	0.021	Ct.vBMD	β-CTX	-0.292	0.005	0.085	8.197	0.005
BV/TV	BMI	0.189	0.048	0.315	7.742	0	BV/TV	Age	-0.236	0.016	0.215	7.83	0
	Age	-0.263	0.005					WC/HC	-0.315	0.002			
	Diabetes	0.23	0.015					BMI	0.304	0.002			
	Sleep efficiency	0.252	0.008				Tb.N	WC/HC	-0.228	0.031	0.052	4.805	0.031
	β-CTX	-0.244	0.011				Tb.Th	BMI	0.508	0	0.232	8.678	0
Tb.N	HC	0.229	0.019	0.248	6.992			Age	-0.306	0.002			
	Ca	-0.341	0.001		0			NC	-0.344	0.008			
	Diabetes	0.299	0.003				Tb.Sp	β-CTX	0.219	0.034	0.095	4.575	0.013
	Age	-0.196	0.047					WC/HC	0.215	0.038			
Tb.Th	BMI	0.272	0.005	0.256	7.324	0	Tb.1/N.SD	β-CTX	0.306	0.003	0.094	9.082	0.003
	Age	-0.237	0.014				Ct.Th	BMI	0.67	0	0.41	14.787	0
	Sleep efficiency	0.232	0.017					NC	-0.51	0			
	Drink	0.217	0.025					Age	-0.284	0.001			
Tb.Sp	HC	-0.208	0.031	0.275	6.375	0		BG	0.24	0.007			
	Diabetes	-0.28	0.004				Ct.Po.Dm	WC	0.22	0.037	0.048	4.476	0.037
	Ca	0.324	0.001										
	Age	0.233	0.018										
	HDL-C	0.192	0.044										
Tb.1/N.SD	TNF-α	0.209	0.026	0.31	6.225	0							
	Ca	0.365	0										
	Diabetes	-0.257	0.007										
	Age	0.214	0.025										
	HDL-C	0.233	0.014										
	WC/HC	0.214	0.023										
Ct.Th	BMI	0.441	0.001	0.186	6.078	0							
	Sleep efficiency	0.235	0.017										
	NC	-0.298	0.023										
	Age	-0.197	0.044										
Ct.Po	Sleep efficiency	0.243	0.016	0.194	5.119	0.001							
	ALP	-0.21	0.039										
	HDL-C	-0.239	0.02										
	25-OHD	0.206	0.048										

*HR-pQCT* high-resolution peripheral quantitative computed tomography, *Tt.Ar* total area, *Ct.Pm* cortical perimeter, *Ct.Ar* cortical area, *Tb.Ar* trabecular area, *BMD* bone mineral density, *Tt.vBMD* total volumetric vBMD, *Tb.vBMD* trabecular vBMD, *Ct.vBMD* cortical vBMD, *BV/TV* trabecular bone volume fraction, *Tb.N* trabecular number, *Tb.Th* trabecular thickness, *Tb.Sp* trabecular separation, *Tb.1/N.SD* trabecular bone heterogeneity, *Ct.Th* cortical thickness, *Ct.Po* intra-cortical porosity, *Ct.Po.Dm* cortical pore diameter, *NC* neck circumference, *WC* waist circumference, *HC* hip circumference, *BMI* body mineral index, *HDL-C* serum high-density lipoprotein cholesterol, *P* Phosphorus, *β-CTX* β-C-terminal telopeptide of type I collagen, *Ca* calcium, *TG* triglycerides, *TNF-α* Tumor necrosis factor, *ALP* alkaline phosphatase, *BG* blood glucose, *25-OHD* total 25- hydroxyvitamin D

## Discussion

This study showed the vBMD, Tb.Th and Ct.Th of OSA patients were lower than non-OSA group. The negative impact of OSA on bone had already appeared in middle-aged patients with OSA. The mechanisms underlying the above phenomenon remain unclear, and might be related to factors including body shape, metabolic complications, changes in sleep efficiency, and sleep-related hypoxemia.

There was no significant difference in HR-pQCT parameters of radius among the four groups of people, but the difference in tibia was more obvious. A possible reason might be that body weight has a greater effect on lower limb bones than upper ones. In terms of bone geometric parameters, the difference between the four groups of people was mainly shown in Ct.Ar. Ct.Ar in the severe OSA group was higher than mild and moderate OSA groups. Considering the difference in BMI of the four groups of patients, the correlation and regression analysis results, the result was mainly due to the larger BMI in the severe OSA group. The difference in vBMD was mainly reflected in Tt.vBMD and Tb.Meta.vBMD. These indicators in OSA patients were much lower than the normal population, which was consistent with some previous studies [6, 38]. The difference in bone microstructure was mainly reflected in Tb.Th and Ct.Th. The result was consistent with a previous meta-analysis which also showed that Ct.vBMD, Tb.Th and stiffness were better predictors for osteoporosis and fragility fractures compared with other HR-qPCT parameters. Despite OSA patients had lower levels, a correlation and regression analysis showed that these two indicators were only related to demographics like age, BMI, and neck circumference. In addition, a regression analysis showed that sleep efficiency was linearly correlated with multiple parameters of HR-pQCT. Therefore, we believe that changes in sleep efficiency in patients with OSA might have a negative impact on bone quality to some extent. There have also been several reports of bone loss caused by abnormal sleep [17, 39], which was consistent with the results of this study.

To date, there have only been a few studies regarding the correlation between OSA and osteoporosis, and conclusions remain controversial. Some studies have suggested negative association. Zhao et al. also conducted a similar study in young male participants. They found that moderate OSA patients had higher BMD at lumbar spine than control and severe OSA patients. In 2012, Mariani recruited 115 obese OSA patients (56 men, 59 women, BMI 30–40 kg/m^2^) for a cross-sectional study, using DEXA to measure the BMD of their lumbar spine, total iliac, and femoral neck. They found no difference in BMD among OSA patients across different severities, and the regression analysis did not find a clear correlation between AHI and BMD [21]. However, it is worth noting that the study did not have a control group. The above research conclusions were consistent with those of Torres et al., who exposed orchiectomized mice to intermittent hypoxia to simulate OSA but did not find a difference in femoral trabecular BMD with the control group [11]. Although the author presupposed that 32 days exposure was sufficient to observe the changes, this may differ from the actual situation of human beings. Some studies have suggested that OSA has a positive effect on bone metabolism. Using abdominal CT, Daniel et al. observed decreased BMD in OSA patients after controlling for age, gender, and cardiovascular diseases. In 2013, Sforza recruited 832 elderly patients with OSA. Using DEXA, they found that the BMD of their femurs and spine were higher than those of healthy controls. BMI, AHI, and HI were closely related to BMD. They believed that intermittent hypoxemia could promote bone remodeling in the elderly. The research subjects were limited to the elderly and cannot be inferred to the entire population [8]. In 2017, Chen recruited 71 patients with OSA and 13 controls. Using DEXA, they found that the BMD and t-values ​​of the hips of OSA patients were higher than the control group. As the severity of OSA increased, blood adiponectin decreased, and the level of blood adiponectin was negatively correlated with total hip BMD. They concluded that changes in plasma adiponectin levels might be one of the reasons for which OSA affects BMD [40]. However, most current studies believed that OSA had a negative effect on bone quality. In 2016, Hamada evaluated 234 study subjects (180 men and 54 women) by CT and found that the lumbar spine BMD of patients with severe OSA was lower. However, a correlation analysis showed that in males, BMD was mainly affected by age, hypertension, and alveolar arterial oxygen differential pressure, while females were mainly affected by age. In this study, since the control group had a higher ESS score, the possibility of combining undetected sleep diseases should be considered [38]. Uzkeser et al. showed that compared with the control group, 21 OSA patients had a higher risk of osteoporosis. The lumbar t-score, BMD, and femoral neck BMD were significantly lower than the control group [6]. The results of a large-sample cohort study in Taiwan in 2012 showed that the risk of osteoporosis in OSA patients was 2.74 times that of non-OSA patients. However, the control group might be mixed with undiagnosed OSA patients, and the influence of weight and age on BMD cannot be completely ruled out [9]. The contradictive results in the above research may be due to confounders controlling and different study designs.

In this study, the four groups of patients showed no significant differences in inflammation indicators. However, it has been reported that OSA patients can increase the level of systemic inflammatory factors such as IL-6, TNF-α, and CRP. This might interfere with normal bone metabolism [14, 41]. However, in our study, we found no increase in any inflammatory factors, which may be related to the small sample size.

No differences in blood bone turnover indicators were found between the groups in this study. This may be attributed to younger subjects recruited who may not yet show any significant changes. In 2017, Chen recruited 71 OSA patients and 13 control groups to test their blood bone turnover indicators, but found no significant difference [40]. Tomiyama found that 50 patients with OSA had a significant increase in urine CTX, and further discovered that 21 patients with OSA had a decrease in urine CTX after CPAP treatment [5]. A recent study confirmed that CPAP treatment could improve BMD, vitamin D and Ca levels in male OSA patients. In the study of Terzi, the average femoral neck BMD of 30 OSA patients was lower than that of the control group, and the serum β-CTX level was higher [7]. The researchers believed that OSA could promote bone resorption to some extent. However, other bone turnover indicators such as Ca, P, bone-specific ALP, and T-25OH, did not show statistical differences. Erden found that compared with the control group, the OSA group had higher PTH and lower T-25OHD levels [42]. In our study, the correlation analysis showed that HI and %TST-SpO_2_ < 90% were positively correlated with ALP, a common osteogenic indicator, which suggest that hypoxia in OSA might interfere with the process of bone formation. However, total ALP was influenced by many factors and bone-specific ALP was not collected in this study, the conclusion needs further verification.

This study had several advantages. First, this study evaluates the bone condition of OSA patients by HR-pQCT for the first time. No study has reported changes in bone geometric parameters and bone microstructure in patients with OSA. Second, the PSG used in this study is more accurate than the portable ones, and HR-pQCT is more reasonable than DEXA. In addition, this study has strict controls on confounding factors. However, limitations exist in the current study. The cross-sectional design can only assess the correlation between OSA and osteoporosis. Some important confounders, such as nutritional status and exercise amount, which would affect bone metabolism, were not evaluated. In addition, the sample size was relatively small. Women and elderly were not included in this study. Both OSA and osteoporosis are affected by gender, age and menopausal status. The well-established feedback regulation in young and male participants might be the reason for insignificant findings in regression analysis. Finally, the inconsistent result in pairwise comparison suggests complex mechanisms behind these two diseases, which needs to be interpreted with caution.

## Conclusions

This study found that OSA might negatively impact the osteogenesis process. It manifested as a decrease in vBMD, tibial cortex, and bone trabecular thickness; this change has already appeared around middle age. These abnormalities could be the potential indicators for CPAP treatment which needs further validation. The specific mechanism remains unclear but may be related to factors such as body shape, metabolic complications, changes in sleep efficiency, and sleep-related hypoxemia. A study with a larger sample is necessary to further assess the relationship and mechanisms between OSA and osteoporosis.

### Supplementary Information

**Additional file1:** **Table S1.** Pairwise comparison of general clinical data and polysomnographic parameters between OSA and control group1 subjects. **Table S2.** Pairwise comparison of blood test results between OSA and control group subjects. **Table S3.** HR-pQCT parameters of radius of OSA and control group subjects. **Table S4.** Pairwise comparison of HR-pQCT parameters of OSA and control group subjects. **Table S5.** Correlation analysis of general information and blood test results. **Table S6.** Correlation analysis of general clinical data and radius HR-pQCT parameters. **Table S7.** Correlation analysis of general clinical data and tibia HR-pQCT parameters. **Table S8.** Correlation analysis of peripheral blood indexes and radius HR-pQCT parameters. **Table S9.** Correlation analysis of peripheral blood indexes and tabia HR-pQCT parameters.

## Data Availability

The data that support the findings of this study are available from the corresponding author upon reasonable request.

## Abbreviations

ALP: Alkaline phosphatase
AHI: Apnea hypopnea index
β-CTX: β-I collagen carboxy-terminal peptide
BG: Blood glucose
BMD: Bone mineral density
BMI: Body mass index
BV/TV: Trabecular bone volume fraction
CI: Confidence interval
CO_2_: Carbon dioxide
COPD: Chronic obstructive pulmonary disease
CPAP: Continuous positive airway pressure
Ct.Ar: Cortical area
Ct.Pm: Cortical perimeter
Ct.Po: Intra-cortical porosity
Ct.Po.Dm: Cortical pore diameter
Ct.Th: Cortical thickness
Ct.vBMD: Cortical vBMD
DEXA: Dual energy X-ray absorptiometry
ESR: Erythrocyte sedimentation rate
ESS: Epworth sleepiness scale
HDL-C: High-density lipoprotein cholesterol
HR-pQCT: High-resolution peripheral quantitative computer tomography
HsCRP: High-sensitivity C-reactive protein
IL-6: Interleukin-6
ODI: Oxygen desaturation index
OR: Odds ratio
OSA: Obstructive sleep apnea
PSG: Polysomnography
SpO_2_: Oxygen saturation
% TST- SpO_2_ < 90%: Percentage of TST with SpO_2_ < 90%
T-25OHD: Total 25-hydroxyvitamin D
Tb.1/N.SD: Trabecular bone heterogeneity
Tb.Ar: Trabecular area
Tb.Inn.vBMD: Internal trabecular vBMD
Tb.Meta.vBMD: External trabecular vBMD
Tb.N: Trabecular number
Tb.Sp: Trabecular separation
Tb.Th: trabecular thickness
Tb.vBMD: Trabecular vBMD
TC: Total cholesterol
TG: Triglycerides
TNF-α: Tumor necrosis factor- α
TST: Total sleep time
Tt.Ar: Total area
Tt.vBMD: Total volumetric vBMD
vBMD: Volumetric bone mineral density
