# Supplementary material for: Early bone loss in patients with obstructive sleep apnea: a cross-sectional study
Source: BMC Pulm Med. 2024 Jan 11;24:28. doi: 10.1186/s12890-024-02848-7 (PMC10782667; doi:10.1186/s12890-024-02848-7)
Supplement: Supplementary file 1 — Additional file1: Table S1. Pairwise comparison of general clinical data and polysomnographic parameters between OSA and control group1 subjects. Table S2. Pairwise comparison of blood test results between OSA and control group subjects. Table S3. HR-pQCT parameters of radius of OSA and control group subjects. Table S4. Pairwise comparison of HR-pQCT parameters of OSA and control group subjects. Table S5. Correlation analysis of general information and blood test results. Table S6. Correlation analysis of general clinical data and radius HR-pQCT parameters. Table S7. Correlation analysis of general clinical data and tibia HR-pQCT parameters. Table S8. Correlation analysis of peripheral blood indexes and radius HR-pQCT parameters. Table S9. Correlation analysis of peripheral blood indexes and tabia HR-pQCT parameters. [file 12890_2024_2848_MOESM1_ESM.docx]

| Table S1. Pairwise comparison of general clinical data and polysomnographic parameters between OSA and control group1 subjects. | | | | | |
| --- | --- | --- | --- | --- | --- |
| Items |  | Control group | Mild OSA group | Moderate OSA group | Severe OSA group |
| BMI,kg/m^2^ | Control group |  | 1.000 | 1.000 | 0.036 |
|  | Mild OSA group | 1.000 |  | 1.000 | 0.021 |
|  | Moderate OSA group | 1.000 | 1.000 |  | 0.076 |
|  | Severe OSA group | 0.036 | 0.021 | 0.076 |  |
| AHI, /h | Control group |  | 0.440 | 0.000 | 0.000 |
|  | Mild OSA group | 0.440 |  | 0.018 | 0.000 |
|  | Moderate OSA group | 0.000 | 0.018 |  | 0.001 |
|  | Severe OSA group | 0.000 | 0.000 | 0.001 |  |
| AI, /h | Control group |  | 0.247 | 0.002 | 0.000 |
|  | Mild OSA group | 0.247 |  | 0.231 | 0.000 |
|  | Moderate OSA group | 0.002 | 0.231 |  | 0.001 |
|  | Severe OSA group | 0.000 | 0.000 | 0.001 |  |
| HI, /h | Control group |  | 0.164 | 0.001 | 0.000 |
|  | Mild OSA group | 0.164 |  | 0.127 | 0.006 |
|  | Moderate OSA group | 0.001 | 0.127 |  | 1.000 |
|  | Severe OSA group | 0.000 | 0.006 | 1.000 |  |
| Lowest SpO_2_,% | Control group |  | 1.000 | 0.144 | 0.000 |
|  | Mild OSA group | 1.000 |  | 0.527 | 0.000 |
|  | Moderate OSA group | 0.144 | 0.527 |  | 0.003 |
|  | Severe OSA group | 0.000 | 0.000 | 0.003 |  |
| Average SpO_2_,% | Control group |  | 1.000 | 0.821 | 0.004 |
|  | Mild OSA group | 1.000 |  | 0.450 | 0.000 |
|  | Moderate OSA group | 0.821 | 0.450 |  | 0.110 |
|  | Severe OSA group | 0.004 | 0.000 | 0.110 |  |
| % TST- SpO_2_ < 90%,% | Control group |  | 1.000 | 0.359 | 0.000 |
|  | Mild OSA group | 1.000 |  | 0.279 | 0.000 |
|  | Moderate OSA group | 0.359 | 0.279 |  | 0.001 |
|  | Severe OSA group | 0.000 | 0.000 | 0.001 |  |
| OSA, obstructive sleep apnea; BMI, body mass index; ESS, Epworth Sleep Scale; AHI, apnea-hypopnea index; AI, apnea index; HI, hypopnea index; SpO_2_, oxygen saturation; % TST- SpO_2_ < 90%, percentage of TST with SpO_2_ < 90%. | | | | | |

| Table S2. Pairwise comparison of blood test results between OSA and control group subjects. | | | | | |
| --- | --- | --- | --- | --- | --- |
| Items |  | Control group1 | Mild OSA group | Moderate OSA group | Severe OSA group |
| TG | Control group | | 0.058 | 0.075 | 0.009 |
|  | Mild OSA group | 0.058 |  | 1.000 | 1.000 |
|  | Moderate OSA group | 0.075 | 1.000 |  | 1.000 |
|  | Severe OSA group | 0.009 | 1.000 | 1.000 |  |
| BG | Control group | | 1.000 | 1.000 | 1.000 |
|  | Mild OSA group | 1.000 |  | 0.513 | 0.006 |
|  | Moderate OSA group | 1.000 | 0.513 |  | 1.000 |
|  | Severe OSA group | 1.000 | 0.006 | 1.000 |  |
| OSA, obstructive sleep apnea; TG, triglycerides; BG, blood sugar. | | | | | |

| Table S3. HR-pQCT parameters of radius of OSA and control group subjects. | | | | | | | | |
| --- | --- | --- | --- | --- | --- | --- | --- | --- |
| Items |  | Total | Control group | mild OSA group | Moderate OSA group | Severe OSA group | *F/χ^2^*-value | *P*-value |
| geometric | Tt.Ar（mm^2^） | 344.46±50.10 | 324.08±54.68 | 340.19±52.01 | 346.23±54.89 | 337.10±44.17 | 0.911 | 0.439 |
| parameters | Ct.Pm（mm） | 78.85(74.40-83.20） | 78.35(72.15-81.70) | 76.90(74.20-82.90) | 78.20(74.50-86.10) | 80.60(76.13-83.78) | 3.452 | 0.331 |
|  | Ct.Ar（mm^2^） | 79.28±12.22 | 79.34±15.59 | 76.46±11.33 | 76.24±10.96 | 83.21±11.92 | 2.128 | 0.103 |
|  | Tb.Ar（mm^2^） | 269.43±51.11 | 248.86±55.74 | 267.92±49.45 | 274.26±58.55 | 273.62±46.67 | 0.679 | 0.568 |
| BMD | Tt.vBMD（mg HA/ccm） | 334.17±62.81 | 350.12±75.80 | 320.74±62.48 | 330.07±67.46 | 341.89±56.49 | 0.791 | 0.502 |
|  | Tb.vBMD（mg HA/ccm） | 164.54±37.17 | 168.27±38.26 | 152.81±42.92 | 169.53±35.69 | 168.99±32.71 | 1.158 | 0.331 |
|  | Tb.Meta.vBMD（mg HA/ccm） | 226.38±38.08 | 232.32±36.29 | 211.50±42.07 | 231.55±36.32 | 232.38±35.11 | 1.812 | 0.151 |
|  | Tb.Innn.vBMD（mg HA/ccm） | 122.29±37.86 | 124.50±40.19 | 112.72±44.72 | 127.19±36.88 | 125.67±32.39 | 0.745 | 0.528 |
|  | Ct.vBMD（mg HA/ccm） | 913.20(874.75-940.00） | 923.35(840.75-954.45) | 913.50(883.20-946.20) | 895.90(872.60-930.90) | 911.90(874.20-939.10) 0.907 | | 0.824 |
| microstructure | BV/TV | 0.24±0.05 | 0.25±0.22 | 0.23±0.06 | 0.25±0.05 | 0.25±0.05 | 1.142 | 0.337 |
| parameters | Tb.N（/mm） | 1.45±0.21 | 1.51±0.21 | 1.42±0.25 | 1.47±0.17 | 1.45±0.21 | 0.501 | 0.682 |
|  | Tb.Th（mm） | 0.24±0.02 | 0.24±0.02 | 0.24±0.02 | 0.24±0.02 | 0.25±0.02 | 1.754 | 0.162 |
|  | Tb.Sp（mm） | 0.65(0.60-0.72） | 0.64(0.58-0.70) | 0.67（0.60-0.79) | 0.62（0.61-0.70) | 0.65（0.59-0.74) | 2.172 | 0.537 |
|  | Tb.1/N.SD（mm） | 0.26(0.24-0.30） | 0.26(0.22-0.28) | 0.27(0.24-0.30) | 0.25(0.23-0.27) | 0.27(0.23-0.30) | 3.272 | 0.351 |
|  | Ct.Th（mm） | 1.20±0.19 | 1.23±0.24 | 1.16±0.15 | 1.15±0.21 | 1.25±0.19 | 1.602 | 0.195 |
|  | Ct.Po | 0.01(0.00-0.01） | 0.01(0.00-0.01) | 0.01(0.01-0.01) | 0.01(0.01-0.01） | 0.01(0.00-0.01) | 1.583 | 0.663 |
|  | Ct.Po.Dm（mm） | 0.18(0.16-0.19) | 0.17(0.17-0.20 | 0.17(0.16-0.19) | 0.18(0.17-0.20) | 0.18(0.16-0.20) | 1.288 | 0.732 |
| HR-pQCT,high-resolution peripheral quantitative computed tomography;OSA,obstructive sleep apnea; Tt.Ar,total area;Ct.Pm,cortical perimeter;Ct.Ar,cortical area;Tb.Ar,trabecular area; BMD, bone mineral density;Tt.vBMD,total volumetric vBMD;Tb.vBMD,trabecular vBMD;Tb.Meta.vBMD,external trabecular vBMD;Tb.Inn.vBMD, internal trabecular vBMD;Ct.vBMD,cortical vBMD;BV/TV,trabecular bone volume fraction;Tb.N,trabecular number;Tb.Th,trabecular thickness;Tb.Sp,trabecular separation;Tb.1/N.SD,trabecular bone heterogeneity;Ct.Th,cortical thickness;Ct.Po,intra-cortical porosity;Ct.Po.Dm,cortical pore diameter. | | | | | | | | |

| Table S4. Pairwise comparison of HR-pQCT parameters of OSA and control group subjects. | | | | | | |
| --- | --- | --- | --- | --- | --- | --- |
| Items |  | Groups | Control group | mild OSA group | Moderate OSA group | Severe OSA group |
| Tibia | Ct.Ar, | Control group |  | 0.398 | 0.910 | 1.000 |
|  |  | Mild OSA group | 0.298 |  | 1.000 | 0.006 |
|  |  | Moderate OSA group | 0.910 | 1.000 |  | 0.048 |
|  |  | Severe OSA group | 1.000 | 0.006 | 0.048 |  |
|  | Tt.vBMD | Control group |  | 0.141 | 0.290 | 1.000 |
|  |  | Mild OSA group | 0.141 |  | 1.000 | 0.172 |
|  |  | Moderate OSA group | 0.290 | 1.000 |  | 0.475 |
|  |  | Severe OSA group | 1.000 | 0.172 | 0.475 |  |
|  | Tb.Meta.vBMD | Control group |  | 0.025 | 0.270 | 1.000 |
|  |  | Mild OSA group | 0.025 |  | 1.000 | 0.089 |
|  |  | Moderate OSA group | 0.270 | 1.000 |  | 1.000 |
|  |  | Severe OSA group | 1.000 | 0.089 | 1.000 |  |
|  | Tb.Th | Control group |  | 0.001 | 0.001 | 0.059 |
|  |  | Mild OSA group | 0.001 |  | 1.000 | 0.295 |
|  |  | Moderate OSA group | 0.001 | 1.000 |  | 0.147 |
|  |  | Severe OSA group | 0.059 | 0.295 | 0.147 |  |
|  | Ct.Th | Control group |  | 0.049 | 0.091 | 1.000 |
|  |  | Mild OSA group | 0.049 |  | 1.000 | 0.026 |
|  |  | Moderate OSA group | 0.091 | 1.000 |  | 0.073 |
|  |  | Severe OSA group | 1.000 | 0.026 | 0.073 |  |
| OSA, obstructive sleep apnea; Ct.Ar, cortical bone area; Tt.vBMD, total volume bone density; Tb.Meta.vBMD, external cancellous bone volume bone density; Tb.Th, trabecular bone thickness; Ct. Th, the thickness of cortical bone. | | | | | | |

| Table S5. Correlation analysis of general information and blood test results. | | | | | | | | | | | | | |
| --- | --- | --- | --- | --- | --- | --- | --- | --- | --- | --- | --- | --- | --- |
| Items | TC | TG | HDL-C | BG | IL-6 | TNF-α | ESR | HsCRP | Ca | P | T-25OHD | β-CTX | ALP |
| Age,year | -0.148 | -0.131 | 0.012 | 0.167 | 0.028 | 0.077 | -0.090 | -0.105 | -0.193 | -0.126 | 0.164 | 0.020 | 0.007 |
| BMI,kg/m^2^ | -0.108 | 0.085 | -0.214 | 0.154 | 0.087 | -0.051 | -0.110 | 0.195 | 0.120 | 0.037 | 0.008 | -0.152 | 0.018 |
| SBP,mmHg | 0.129 | 0.190 | 0.024 | 0.002 | -0.151 | -0.110 | 0.145 | 0.221 | 0.063 | 0.710 | 0.748 | -0.138 | -0.021 |
| DBP,mmHg | 0.027 | 0.126 | -0.020 | -0.038 | -0.280^**^ | -0.137 | 0.138 | 0.079 | 0.164 | -0.044 | 0.068 | -0.264^*^ | -0.027 |
| MBP,mmHg | 0.099 | 0.159 | -0.004 | -0.001 | -0.258^*^ | -0.157 | 0.159 | 0.148 | 0.164 | -0.043 | 0.067 | -0.253^*^ | -0.030 |
| NC,cm | -0.047 | 0.162 | -0.156 | 0.178 | -0.044 | -0.077 | -0.150 | 0.061 | 0.097 | 0.031 | -0.088 | -0.080 | 0.041 |
| WC,cm | -0.096 | 0.177 | -0.184 | 0.219^*^ | 0.102 | -0.026 | 0.081 | 0.128 | 0.024 | -0.002 | -0.134 | -0.044 | 0.187 |
| HC,cm | -0.061 | -0.025 | -0.097 | 0.274^**^ | -0.062 | -0.232^*^ | -0.033 | 0.03 | 0.066 | 0.006 | -0.070 | -0.056 | 0.172 |
| WC/HC | -0.015 | 0.279^**^ | -0.157 | -0.047 | 0.192 | 0.249^*^ | 0.139 | 0.137 | -0.073 | 0.083 | -0.089 | 0.039 | 0.142 |
| ESS | -0.001 | 0.229^*^ | -0.246^*^ | 0.148 | 0.068 | -0.060 | 0.055 | 0.006 | 0.038 | 0.025 | -0.085 | 0.075 | -0.096 |
| Hypertension,% | -0.032 | 0.058 | 0.113 | 0.052 | -0.222^*^ | -0.020 | 0.145 | 0.095 | 0.038 | -0.025 | 0.023 | -0.221^*^ | -0.084 |
| Diabetes,% | -0.138 | -0.096 | -0.066 | 0.410^**^ | -0.128 | 0.071 | 0.164 | -0.027 | 0.144 | -0.160 | 0.067 | -0.093 | 0.052 |
| Dyslipidemia,% | 0.277^**^ | 0.742^**^ | -0.286^**^ | 0.061 | 0.056 | -0.177 | 0.096 | 0.100 | 0.180 | 0.032 | -0.057 | 0.062 | 0.166 |
| Smoking history,% | -0.011 | 0.106 | -0.095 | -0.097 | 0.009 | 0.004 | 0.018 | -0.052 | -0.076 | 0.010 | -0.157 | 0.041 | 0.078 |
| drinking history,% | -0.069 | 0.141 | -0.137 | -0.004 | -0.132 | -0.043 | 0.136 | -0.196 | -0.078 | -0.088 | 0.018 | 0.098 | -0.018 |
| AHI,/h | 0.025 | 0.225^*^ | -0.115 | 0.281^**^ | 0.021 | 0.025 | 0.131 | 0.099 | 0.057 | 0.009 | 0.010 | 0.067 | 0.186 |
| AI,/h | 0.017 | 0.176 | -0.054 | 0.292^**^ | 0.040 | 0.059 | 0.118 | 0.051 | 0.061 | -0.019 | 0.024 | 0.050 | 0.146 |
| HI,/h | 0.057 | 0.188 | -0.145 | 0.143 | 0.042 | 0.030 | 0.072 | 0.127 | -0.006 | -0.028 | 0.016 | 0.048 | 0.213^*^ |
| Lowest SpO_2_,% | 0.108 | -0.191 | 0.086 | -0.192 | -0.092 | -0.111 | -0.063 | -0.100 | -0.082 | -0.116 | 0.027 | -0.004 | -0.157 |
| Average SpO_2_,% | 0.104 | -0.176 | 0.210^*^ | -0.096 | -0.110 | -0.088 | -0.081 | -0.154 | -0.101 | -0.184 | -0.004 | -0.143 | -0.201 |
| % TST- SpO_2_ < 90%,% | -0.090 | 0.170 | -0.105 | 0.156 | 0.128 | 0.123 | 0.099 | 0.137 | 0.090 | 0.176 | -0.068 | -0.002 | 0.212^*^ |
| Sleep efficiency | 0.072 | 0.103 | -0.077 | -0.099 | -0.028 | 0.173 | -0.097 | -0.037 | -0.048 | 0.106 | -0.177 | 0.121 | 0.015 |
| TC | 1.000 | 0.178 | 0.144 | 0.073 | -0.050 | -0.203 | -0.016 | 0.110 | 0.237^*^ | 0.177 | 0.135 | 0.228^*^ | 0.212^*^ |
| TG | 0.178 | 1.000 | -0.561^**^ | 0.080 | 0.013 | -0.116 | 0.183 | 0.087 | 0.267^*^ | 0.101 | -0.104 | 0.091 | 0.249^*^ |
| HDL-C | 0.144 | -0.561^**^ | 1.000 | -0.063 | -0.101 | 0.063 | -0.167 | -0.057 | -0.137 | 0.093 | 0.169 | 0.077 | -0.147 |
| BG | 0.073 | 0.080 | -0.063 | 1.000 | -0.120 | -0.255^*^ | 0.070 | -0.116 | 0.112 | -0.158 | 0.018 | -0.042 | 0.140 |
| IL-6 | -0.050 | 0.013 | -0.101 | -0.120 | 1.000 | 0.298^**^ | -0.003 | 0.224^*^ | -0.079 | 0.149 | -0.166 | -0.038 | -0.110 |
| TNF-α | -0.203 | -0.116 | 0.063 | -0.255^*^ | 0.298^**^ | 1.000 | 0.070 | -0.005 | 0.038 | 0.027 | 0.018 | 0.109 | -0.063 |
| ESR | -0.016 | 0.183 | -0.167 | 0.070 | -0.003 | 0.070 | 1.000 | 0.243^*^ | 0.138 | 0.228^*^ | 0.081 | 0.038 | 0.160 |
| HsCRP | 0.110 | 0.087 | -0.057 | -0.116 | 0.224^*^ | -0.005 | 0.243^*^ | 1.000 | 0.237^*^ | 0.153 | 0.042 | -0.001 | 0.192 |
| Ca | 0.237^*^ | 0.267^*^ | -0.137 | 0.112 | -0.079 | 0.038 | 0.138 | 0.237^*^ | 1.000 | 0.138 | 0.217^*^ | 0.102 | 0.183 |
| P | 0.177 | 0.101 | 0.093 | -0.158 | 0.149 | 0.027 | 0.228^*^ | 0.153 | 0.138 | 1.000 | -0.040 | 0.099 | 0.081 |
| T-25OHD | 0.135 | -0.104 | 0.169 | 0.018 | -0.166 | 0.018 | 0.081 | 0.042 | 0.217^*^ | -0.040 | 1.000 | 0.203 | -0.194 |
| β-CTX | 0.228^*^ | 0.091 | 0.077 | -0.042 | -0.038 | 0.109 | 0.038 | -0.001 | 0.102 | 0.099 | 0.203 | 1.000 | 0.300^**^ |
| ALP | 0.212^*^ | 0.249^*^ | -0.147 | 0.140 | -0.110 | -0.063 | 0.160 | 0.192 | 0.183 | 0.081 | -0.194 | 0.300^**^ | 1.000 |
| **P＜0.01，*P＜0.05 | | | | | | | | | | | | | |

BMI, body mass index; SBP, systolic blood pressure;DBP,diastolic blood pressure; MBP,mean blood pressure;NC, neck circumference; WC, waist circumference;HC, hip circumference；ESS, Epworth Sleep Scale;AHI, apnea-hypopnea index; AI, apnea index; HI, hypopnea index; SpO_2_, oxygen saturation;TST, total sleep time; % TST- SpO_2_ < 90%, percentage of TST with SpO_2_ < 90%; TC,cholesterol; TG,triglycerides; HDL-C,high-density lipoprotein cholesterol; BG,blood glucose; IL-6,interleukin-6; TNF-α,tumor necrosis factor- α; ESR,erythrocyte sedimentation rate; HsCRP,high-sensitivity C-reactive protein; Ca, calcium; P,blood phosphorus; T-25OHD,total 25-hydroxyvitamin D; β-CTX,β-I collagen carboxy-terminal peptide; ALP,alkaline phosphatase. 1mmHg=0.133kpa.

Table S6. Correlation analysis of general clinical data and radius HR-pQCT parameters.

| Items | Tt.Ar | Ct.Pm | Ct.Ar | Th.Ar | Tt.vBMD | Tb.vBMD | Tb.Meta.vBMD | Tb.Inn.vBMD | Ct.vBMD | BV比TV | Tb.N | Tb.Th | Tb.Sp | Tb.1比N.SD | Ct.Th | Ct.Po | Ct.Po.Dm |
| --- | --- | --- | --- | --- | --- | --- | --- | --- | --- | --- | --- | --- | --- | --- | --- | --- | --- |
| Age | 0.155 | 0.179 | -0.148 | 0.188 | -0.287^**^ | -0.261^*^ | -0.227^*^ | -0.275^**^ | -0.233^*^ | -0.278^**^ | -0.135 | -0.289^**^ | 0.146 | 0.152 | -0.205 | 0.196 | -0.046 |
| BMI | 0.199 | 0.192 | 0.393^**^ | 0.103 | 0.220^*^ | 0.302^**^ | 0.275^**^ | 0.311^**^ | -0.001 | 0.316^**^ | 0.242^*^ | 0.311^**^ | -0.261* | -0.150 | 0.284^**^ | 0.100 | 0.093 |
| SBP | 0.044 | -0.031 | -0.086 | 0.064 | -0.050 | -0.013 | -0.023 | -0.005 | 0.033 | -0.011 | -0.067 | 0.026 | 0.071 | 0.112 | -0.067 | 0.037 | 0.096 |
| DBP | -0.106 | -0.104 | -0.075 | -0.071 | 0.012 | 0.030 | 0.036 | 0.007 | -0.017 | 0.033 | 0.020 | 0.056 | -0.013 | 0.046 | -0.044 | 0.005 | -0.023 |
| MBP | -0.095 | -0.092 | -0.059 | -0.069 | 0.028 | 0.038 | 0.031 | 0.024 | 0.013 | 0.040 | -0.008 | 0.074 | 0.007 | 0.065 | -0.017 | 0.012 | 0.019 |
| NC | 0.264^*^ | 0.244^*^ | 0.143 | 0.223^*^ | -0.025 | 0.142 | 0.122 | 0.150 | -0.167 | 0.149 | 0.107 | 0.173 | -0.097 | -0.040 | 0.009 | 0.042 | -0.041 |
| WC | 0.152 | 0.163 | 0.192 | 0.104 | 0.064 | 0.163 | 0.165 | 0.156 | -0.060 | 0.169 | 0.148 | 0.159 | -0.123 | -0.046 | 0.128 | -0.020 | -0.046 |
| HC | 0.199 | 0.162 | 0.195 | 0.149 | 0.121 | 0.299^**^ | 0.289^**^ | 0.297^**^ | -0.080 | 0.302^**^ | 0.269^*^ | 0.247^*^ | -0.276^**^ | -0.263^*^ | 0.116 | 0.039 | 0.091 |
| WC/HC | -0.003 | -0.001 | -0.001 | 0.002 | -0.075 | -0.093 | -0.079 | -0.107 | -0.026 | -0.091 | -0.073 | -0.055 | 0.124 | 0.200 | -0.011 | -0.073 | -0.142 |
| ESS | -0.05 | 0.010 | 0.142 | -0.083 | 0.142 | 0.116 | 0.093 | 0.127 | -0.008 | 0.117 | 0.125 | 0.085 | -0.112 | -0.080 | 0.185 | 0.088 | 0.044 |
| Hypertension | -0.136 | -0.079 | 0.050 | -0.152 | 0.136 | 0.097 | 0.065 | 0.115 | 0.056 | 0.107 | 0.099 | 0.078 | -0.091 | -0.005 | 0.103 | 0.036 | 0.088 |
| Diabetes | -0.125 | -0.058 | 0.075 | -0.136 | 0.189 | 0.236^*^ | 0.247^*^ | 0.217^*^ | -0.065 | 0.243^*^ | 0.209^*^ | 0.192 | -0.228^*^ | -0.212^*^ | 0.101 | 0.103 | 0.026 |
| Dyslipidemia | 0.085 | 0.112 | 0.110 | 0.068 | 0.003 | 0.035 | -0.015 | 0.043 | -0.024 | 0.028 | -0.060 | 0.046 | 0.051 | 0.049 | 0.049 | -0.043 | 0.046 |
| Smoking | 0.123 | 0.112 | 0.104 | 0.079 | -0.039 | -0.033 | -0.055 | -0.020 | -0.029 | -0.056 | -0.022 | -0.003 | 0.038 | -0.007 | 0.065 | 0.076 | 0.012 |
| Drinking | -0.017 | -0.068 | 0.069 | -0.023 | 0.053 | 0.139 | 0.135 | 0.130 | -0.029 | 0.145 | 0.030 | 0.213^*^ | -0.041 | -0.002 | 0.038 | 0.089 | -0.015 |
| AHI | 0.115 | 0.159 | 0.182 | 0.065 | 0.113 | 0.158 | 0.146 | 0.164 | 0.000 | 0.166 | 0.037 | 0.210^*^ | -0.053 | 0.028 | 0.153 | -0.024 | -0.028 |
| AI | 0.095 | 0.131 | 0.199 | 0.039 | 0.133 | 0.125 | 0.120 | 0.130 | 0.096 | 0.134 | -0.021 | 0.198 | -0.009 | 0.044 | 0.180 | -0.097 | -0.039 |
| HI | 0.059 | 0.119 | 0.009 | 0.058 | -0.038 | 0.026 | 0.009 | 0.023 | -0.161 | 0.024 | 0.014 | 0.006 | 0.004 | 0.070 | -0.020 | 0.055 | -0.068 |
| Lowest SpO_2_ | -0.037 | -0.069 | -0.063 | -0.004 | -0.006 | -0.016 | -0.011 | -0.031 | 0.024 | -0.027 | 0.054 | -0.143 | -0.048 | -0.110 | -0.064 | 0.007 | 0.018 |
| Average SpO_2_ | -0.025 | -0.054 | -0.088 | 0.015 | -0.041 | -0.056 | -0.035 | -0.070 | 0.070 | -0.060 | -0.085 | -0.139 | 0.061 | -0.020 | -0.103 | -0.084 | -0.023 |
| % TST- SpO_2_ < 90% | 0.016 | 0.049 | 0.150 | -0.033 | 0.084 | 0.085 | 0.078 | 0.097 | 0.001 | 0.091 | -0.012 | 0.185 | -0.006 | 0.044 | 0.144 | 0.030 | 0.050 |
| sleep efficiency | -0.164 | -0.116 | 0.303^**^ | -0.238^*^ | 0.328^**^ | 0.277^**^ | 0.269^*^ | 0.267^*^ | 0.133 | 0.272^**^ | 0.131 | 0.287^**^ | -0.163 | -0.116 | 0.375^**^ | 0.154 | 0.184 |

**P＜0.01，*P＜0.05

HR-pQCT, high-resolution peripheral bone quantitative CT; BMI, body mass index; SBP, systolic blood pressure;DBP,diastolic blood pressure; MBP,mean blood pressure;NC, neck circumference; WC, waist circumference;HC, hip circumference；ESS, Epworth Sleep Scale; AHI, apnea-hypopnea index; AI, apnea index; HI, hypopnea index; TST, total sleep time; % TST- SpO_2_ < 90%, percentage of TST with SpO_2_ < 90%; Tt.Ar,total area; Ct.Pm,cortical perimeter; Ct.Ar,cortical area;Tb.Ar,trabecular area; BMD, bone mineral density; Tt.vBMD,total volumetric vBMD; Tb.vBMD,trabecular vBMD; Tb.Meta.vBMD,external trabecular vBMD; Tb.Inn.vBMD, internal trabecular vBMD; Ct.vBMD,cortical vBMD; BV/TV,trabecular bone volume fraction; Tb.N,trabecular number; Tb.Th,trabecular thickness; Tb.Sp,trabecular separation; Tb.1/N.SD,trabecular bone heterogeneity; Ct.Th,cortical thickness; Ct.Po,intra-cortical porosity; Ct.Po.Dm,cortical pore diameter.

Table S7. Correlation analysis of general clinical data and tibia HR-pQCT parameters.

| Items | Tt.Ar | Ct.Pm | Ct.Ar | Th.Ar | Tt.vBMD | Tb.vBMD | Tb.Meta.vBMD | Tb.Inn.vBMD | Ct.vBMD | BV比TV | Tb.N | Tb.Th | Tb.Sp | Tb.1比N.SD | Ct.Th | Ct.Po | Ct.Po.Dm |  |
| --- | --- | --- | --- | --- | --- | --- | --- | --- | --- | --- | --- | --- | --- | --- | --- | --- | --- | --- |
| Age | 0.147 | 0.148 | -0.229^*^ | 0.195 | -0.348^**^ | -0.269^*^ | -0.286^**^ | -0.223^*^ | -0.201 | -0.253^*^ | -0.171 | -0.297^**^ | 0.149 | 0.107 | -0.298^**^ | 0.031 | -0.182 |  |
| BMI | 0.189 | 0.195 | 0.505^**^ | 0.083 | 0.305^**^ | 0.257^*^ | 0.258^*^ | 0.301^**^ | 0.061 | 0.262^*^ | 0.143 | 0.298^**^ | -0.186 | -0.09 | 0.396^**^ | 0.107 | 0.186 |  |
| SBP | -0.043 | -0.042 | 0.086 | -0.061 | 0.118 | 0.061 | 0.085 | 0.070 | 0.065 | 0.059 | -0.013 | 0.061 | -0.019 | -0.034 | 0.133 | 0.080 | 0.019 |  |
| DBP | 0.022 | 0.022 | 0.123 | 0.017 | 0.133 | 0.121 | 0.114 | 0.118 | 0.083 | 0.114 | 0.033 | 0.128 | -0.037 | -0.071 | 0.124 | 0.101 | 0.178 |  |
| MBP | -0.017 | -0.014 | 0.116 | -0.019 | 0.133 | 0.104 | 0.101 | 0.103 | 0.117 | 0.094 | 0.010 | 0.101 | -0.029 | -0.065 | 0.123 | 0.084 | 0.113 |  |
| NC | 0.323^**^ | 0.318^**^ | 0.158 | 0.299^**^ | -0.013 | 0.073 | 0.040 | 0.084 | -0.118 | 0.078 | 0.008 | 0.078 | -0.063 | -0.044 | 0.025 | 0.098 | 0.097 |  |
| WC | 0.162 | 0.157 | 0.178 | 0.124 | 0.021 | 0.043 | 0.015 | 0.081 | -0.024 | 0.042 | -0.009 | 0.072 | -0.023 | -0.044 | 0.087 | 0.092 | 0.188 |  |
| HC | 0.091 | 0.068 | 0.236^*^ | 0.041 | 0.234^*^ | 0.297^**^ | 0.289^**^ | 0.293^**^ | -0.101 | 0.287^**^ | 0.271^**^ | 0.231^*^ | -0.290^**^ | -0.272^**^ | 0.219^*^ | 0.149 | 0.169 |  |
| WC/HC | -0.009 | -0.016 | 0.066 | -0.023 | 0.039 | 0.024 | 0.066 | 0.014 | 0.026 | 0.022 | 0.043 | -0.069 | -0.060 | -0.001 | 0.056 | -0.006 | 0.037 |  |
| ESS | 0.081 | 0.083 | -0.020 | 0.084 | -0.210^*^ | -0.265^*^ | -0.269^*^ | -0.242^*^ | 0.070 | -0.244^*^ | -0.295^**^ | -0.131 | 0.277^**^ | 0.238^*^ | -0.082 | -0.063 | 0.048 |  |
| Hypertension | -0.068 | -0.061 | 0.086 | -0.060 | 0.115 | 0.075 | 0.047 | 0.103 | 0.078 | 0.079 | 0.077 | 0.011 | -0.058 | -0.099 | 0.085 | 0.079 | 0.082 |  |
| Diabetes | 0.014 | 0.027 | -0.013 | 0.010 | 0.062 | 0.147 | 0.114 | 0.177 | -0.142 | 0.148 | 0.148 | 0.069 | -0.140 | -0.157 | 0.007 | 0.132 | 0.018 |  |
| Dyslipidemia | 0.082 | 0.079 | 0.060 | 0.084 | 0.041 | -0.015 | -0.016 | 0.000 | 0.073 | -0.018 | -0.149 | 0.014 | 0.087 | 0.049 | 0.026 | -0.055 | 0.102 |  |
| Smoking | 0.063 | 0.053 | 0.010 | 0.070 | -0.078 | -0.038 | -0.073 | -0.023 | -0.111 | -0.044 | 0.050 | -0.099 | -0.059 | -0.027 | -0.053 | 0.009 | -0.164 |  |
| Drinking | 0.044 | 0.035 | 0.098 | 0.046 | -0.001 | -0.022 | -0.018 | -0.039 | -0.050 | -0.013 | -0.003 | -0.073 | 0.010 | 0.003 | 0.043 | 0.062 | -0.005 |  |
| AHI | 0.123 | 0.139 | 0.261^*^ | 0.091 | 0.149 | 0.098 | 0.118 | 0.090 | 0.069 | 0.107 | 0.108 | 0.040 | -0.123 | -0.009 | 0.155 | 0.014 | 0.043 |  |
| AI | 0.157 | 0.184 | 0.223^*^ | 0.131 | 0.139 | 0.098 | 0.097 | 0.122 | 0.116 | 0.098 | 0.139 | 0.040 | -0.157 | -0.074 | 0.109 | -0.080 | 0.064 |  |
| HI | 0.112 | 0.112 | 0.149 | 0.090 | -0.036 | -0.104 | -0.063 | -0.134 | 0.028 | -0.081 | -0.133 | -0.120 | 0.108 | 0.165 | 0.065 | 0.042 | -0.133 |  |
| Lowest SpO_2_ | 0.020 | -0.001 | -0.157 | 0.041 | -0.078 | 0.002 | -0.051 | 0.022 | -0.047 | -0.022 | 0.031 | -0.058 | -0.024 | -0.136 | -0.104 | 0.021 | -0.035 |  |
| Average SpO_2_ | 0.056 | 0.055 | -0.120 | 0.075 | -0.036 | 0.018 | -0.050 | 0.076 | 0.038 | -0.011 | 0.056 | -0.040 | -0.064 | -0.218^*^ | -0.096 | -0.051 | 0.020 |  |
| % TST- SpO_2_ < 90% | -0.032 | -0.016 | 0.192 | -0.061 | 0.140 | 0.064 | 0.107 | 0.046 | 0.058 | 0.081 | 0.016 | 0.085 | -0.041 | 0.062 | 0.145 | -0.021 | 0.096 |  |
| sleep efficiency | 0.011 | 0.008 | 0.179 | -0.016 | 0.184 | 0.163 | 0.197 | 0.136 | 0.101 | 0.166 | 0.158 | 0.132 | -0.142 | -0.011 | 0.137 | -0.137 | -0.160 |  |
| **P＜0.01，*P＜0.05 | | | | | | | | | | | | | | | | | | |
| HR-pQCT, high-resolution peripheral bone quantitative CT; BMI, body mass index; SBP, systolic blood pressure;DBP,diastolic blood pressure; MBP,mean blood pressure;NC, neck circumference; WC, waist circumference;HC, hip circumference；ESS, Epworth Sleep Scale; AHI, apnea-hypopnea index; AI, apnea index; HI, hypopnea index; TST, total sleep time; % TST- SpO_2_ < 90%, percentage of TST with SpO_2_ < 90%; Tt.Ar,total area; Ct.Pm,cortical perimeter; Ct.Ar,cortical area;Tb.Ar,trabecular area; BMD, bone mineral density; Tt.vBMD,total volumetric vBMD; Tb.vBMD,trabecular vBMD; Tb.Meta.vBMD,external trabecular vBMD; Tb.Inn.vBMD, internal trabecular vBMD; Ct.vBMD,cortical vBMD; BV/TV,trabecular bone volume fraction; Tb.N,trabecular number; Tb.Th,trabecular thickness; Tb.Sp,trabecular separation; Tb.1/N.SD,trabecular bone heterogeneity; Ct.Th,cortical thickness; Ct.Po,intra-cortical porosity; Ct.Po.Dm,cortical pore diameter. | | | | | | | | | | | | | | | | | | |

Table S8. Correlation analysis of peripheral blood indexes and radius HR-pQCT parameters.

| Items | TC | TG | HDL-C | BG | IL-6 | TNF-α | ESR | HsCRP | Ca | P | T-25OHD | β-CTX | ALP |
| --- | --- | --- | --- | --- | --- | --- | --- | --- | --- | --- | --- | --- | --- |
| Tt.Ar | -0.045 | 0.063 | 0.067 | 0.009 | 0.009 | 0.009 | 0.009 | 0.009 | -0.070 | 0.009 | 0.009 | 0.075 | -0.061 |
| Ct.Pm | -0.046 | 0.093 | -0.025 | 0.052 | 0.052 | 0.052 | 0.052 | 0.052 | 0.052 | 0.052 | 0.052 | 0.052 | 0.052 |
| Ct.Ar | -0.120 | 0.016 | -0.139 | 0.060 | 0.060 | 0.060 | 0.060 | 0.060 | 0.049 | 0.060 | 0.060 | -0.170 | -0.064 |
| Th.Ar | -0.008 | 0.055 | 0.107 | 0.004 | 0.004 | 0.004 | 0.004 | 0.004 | -0.081 | 0.004 | 0.004 | 0.114 | -0.045 |
| Tt.vBMD | -0.078 | 0.007 | -0.205 | 0.117 | 0.117 | 0.117 | 0.117 | 0.117 | 0.043 | 0.117 | 0.117 | -0.230^*^ | -0.074 |
| Tb.vBMD | -0.126 | 0.064 | -0.258^*^ | 0.125 | 0.125 | 0.125 | 0.125 | 0.125 | -0.037 | 0.125 | 0.125 | -0.246^*^ | -0.159 |
| Tb.Meta.vBMD | -0.155 | 0.003 | -0.204 | 0.103 | 0.103 | 0.103 | 0.103 | 0.103 | -0.048 | 0.103 | 0.103 | -0.231^*^ | -0.192 |
| Tb.Inn.vBMD | -0.110 | 0.080 | -0.262^*^ | 0.138 | 0.138 | 0.138 | 0.138 | 0.138 | -0.029 | 0.138 | 0.138 | -0.248^*^ | -0.131 |
| Ct.vBMD | 0.008 | -0.075 | -0.045 | 0.007 | 0.007 | 0.007 | 0.007 | 0.007 | 0.007 | 0.007 | 0.007 | 0.007 | 0.007 |
| BV/TV | -0.153 | 0.073 | -0.253^*^ | 0.122 | 0.122 | 0.122 | 0.122 | 0.122 | -0.041 | 0.122 | 0.122 | -0.253^*^ | -0.173 |
| Tb.N | -0.075 | -0.111 | -0.143 | 0.143 | 0.143 | 0.143 | 0.143 | 0.143 | -0.242^*^ | 0.143 | 0.143 | -0.170 | -0.078 |
| Tb.Th | -0.222^*^ | 0.120 | -0.245^*^ | 0.111 | 0.111 | 0.111 | 0.111 | 0.111 | 0.143 | 0.111 | 0.111 | -0.150 | -0.194 |
| Tb.Sp | 0.082 | 0.093 | 0.163 | -0.155 | -0.155 | -0.155 | -0.155 | -0.155 | -0.155 | -0.155 | -0.155 | -0.155 | -0.155 |
| Tb.1比N.SD | 0.038 | 0.146 | 0.106 | -0.152 | -0.152 | -0.152 | -0.152 | -0.152 | -0.152 | -0.152 | -0.152 | -0.152 | -0.152 |
| Ct.Th | -0.062 | 0.025 | -0.137 | 0.085 | 0.085 | 0.085 | 0.085 | 0.085 | 0.113 | 0.085 | 0.085 | -0.138 | -0.009 |
| Ct.Po | -0.207 | 0.040 | -0.141 | -0.101 | -0.101 | -0.101 | -0.101 | -0.101 | -0.101 | -0.101 | -0.101 | -0.101 | -0.101 |
| Ct.Po.Dm | -0.090 | 0.068 | -0.045 | -0.188 | -0.188 | -0.188 | -0.188 | -0.188 | -0.188 | -0.188 | -0.188 | -0.188 | -0.188 |

**P＜0.01，*P＜0.05

HR-pQCT, high-resolution peripheral bone quantitative CT;TC,cholesterol; TG,triglycerides; HDL-C,high-density lipoprotein cholesterol; BG,blood glucose; IL-6,interleukin-6; TNF-α,tumor necrosis factor- α; ESR,erythrocyte sedimentation rate; HsCRP,high-sensitivity C-reactive protein; Ca, calcium; P,blood phosphorus; T-25OHD,total 25-hydroxyvitamin D; β-CTX,β-I collagen carboxy-terminal peptide; ALP,alkaline phosphatase.Tt.Ar,total area; Ct.Pm,cortical perimeter; Ct.Ar,cortical area;Tb.Ar,trabecular area; BMD, bone mineral density; Tt.vBMD,total volumetric vBMD; Tb.vBMD,trabecular vBMD; Tb.Meta.vBMD,external trabecular vBMD; Tb.Inn.vBMD, internal trabecular vBMD; Ct.vBMD,cortical vBMD; BV/TV,trabecular bone volume fraction; Tb.N,trabecular number; Tb.Th,trabecular thickness; Tb.Sp,trabecular separation; Tb.1/N.SD,trabecular bone heterogeneity; Ct.Th,cortical thickness; Ct.Po,intra-cortical porosity; Ct.Po.Dm,cortical pore diameter.

| Table S9. Correlation analysis of peripheral blood indexes and tabia HR-pQCT parameters.   \| Items \| TC \| TG \| HDL-C \| BG \| IL-6 \| TNF-α \| ESR \| HsCRP \| Ca \| P \| T-25OHD \| β-CTX \| ALP \| \| --- \| --- \| --- \| --- \| --- \| --- \| --- \| --- \| --- \| --- \| --- \| --- \| --- \| --- \| \| Tt.Ar \| 0.015 \| 0.067 \| -0.056 \| 0.056 \| -0.122 \| -0.053 \| -0.158 \| 0.102 \| 0.042 \| -0.112 \| 0.015 \| 0.106 \| 0.010 \| \| Ct.Pm \| 0.025 \| 0.070 \| -0.049 \| 0.074 \| -0.097 \| -0.039 \| -0.163 \| 0.097 \| 0.045 \| -0.109 \| -0.004 \| 0.118 \| 0.020 \| \| Ct.Ar \| -0.078 \| 0.072 \| -0.206 \| 0.034 \| -0.105 \| -0.137 \| -0.148 \| -0.016 \| 0.007 \| -0.068 \| -0.036 \| -0.086 \| 0.073 \| \| Th.Ar \| 0.033 \| 0.063 \| -0.012 \| 0.049 \| -0.112 \| -0.042 \| -0.115 \| 0.104 \| 0.041 \| -0.093 \| 0.004 \| 0.123 \| -0.005 \| \| Tt.vBMD \| -0.038 \| 0.041 \| -0.185 \| 0.080 \| -0.112 \| -0.169 \| 0.055 \| 0.047 \| 0.075 \| 0.013 \| 0.006 \| -0.247* \| -0.048 \| \| Tb.vBMD \| -0.069 \| 0.015 \| -0.167 \| 0.104 \| -0.155 \| -0.210* \| 0.114 \| 0.149 \| 0.192 \| 0.068 \| 0.039 \| -0.211* \| -0.090 \| \| Tb.Meta.vBMD \| -0.094 \| 0.044 \| -0.165 \| 0.113 \| -0.147 \| -0.161 \| 0.117 \| 0.162 \| 0.210* \| 0.110 \| 0.065 \| -0.120 \| -0.079 \| \| Tb.Inn.vBMD \| -0.054 \| -0.016 \| -0.118 \| 0.107 \| -0.135 \| -0.236* \| 0.095 \| 0.162 \| 0.143 \| 0.036 \| 0.004 \| -0.285** \| -0.087 \| \| Ct.vBMD \| 0.050 \| -0.036 \| 0.041 \| -0.008 \| -0.019 \| -0.099 \| -0.158 \| -0.107 \| -0.199 \| -0.270* \| 0.059 \| -0.292** \| -0.068 \| \| BV/TV \| -0.075 \| 0.029 \| -0.157 \| 0.098 \| -0.152 \| -0.181 \| 0.121 \| 0.158 \| 0.202 \| 0.080 \| 0.046 \| -0.199 \| -0.090 \| \| Tb.N \| -0.086 \| -0.205 \| 0.030 \| 0.073 \|  \| -0.230* \| 0.002 \| -0.066 \| 0.026 \| 0.017 \| -0.101 \| -0.156 \| -0.094 \| \| Tb.Th \| -0.099 \| 0.087 \| -0.191 \| 0.014 \| -0.039 \| -0.081 \| 0.158 \| 0.184 \| 0.272** \| 0.200 \| 0.129 \| -0.143 \| -0.076 \| \| Tb.Sp \| 0.059 \| 0.140 \| -0.001 \| -0.123 \| 0.224* \| 0.278** \| -0.021 \| 0.069 \| -0.023 \| -0.022 \| 0.114 \| 0.175 \| 0.050 \| \| Tb.1比N.SD \| 0.024 \| 0.172 \| -0.085 \| -0.150 \| 0.246* \| 0.339** \| -0.014 \| 0.130 \| 0.071 \| 0.075 \| 0.120 \| 0.279** \| 0.001 \| \| Ct.Th \| -0.044 \| 0.034 \| -0.196 \| 0.045 \| -0.058 \| -0.121 \| -0.042 \| -0.016 \| 0.035 \| 0.005 \| -0.044 \| -0.107 \| 0.081 \| \| Ct.Po \| -0.126 \| 0.053 \| -0.121 \| 0.059 \| -0.074 \| -0.007 \| 0.085 \| 0.068 \| 0.122 \| 0.119 \| 0.006 \| 0.142 \| 0.024 \| \| Ct.Po.Dm \| -0.134 \| 0.126 \| -0.151 \| 0.085 \| -0.042 \| -0.194 \| -0.047 \| -0.117 \| 0.155 \| -0.064 \| 0.054 \| -0.021 \| 0.078 \| \| **P＜0.01，*P＜0.05  HR-pQCT, high-resolution peripheral bone quantitative CT;TC,cholesterol; TG,triglycerides; HDL-C,high-density lipoprotein cholesterol; BG,blood glucose; IL-6,interleukin-6; TNF-α,tumor necrosis factor- α; ESR,erythrocyte sedimentation rate; HsCRP,high-sensitivity C-reactive protein; Ca, calcium; P,blood phosphorus; T-25OHD,total 25-hydroxyvitamin D; β-CTX,β-I collagen carboxy-terminal peptide; ALP,alkaline phosphatase.Tt.Ar,total area; Ct.Pm,cortical perimeter; Ct.Ar,cortical area;Tb.Ar,trabecular area; BMD, bone mineral density; Tt.vBMD,total volumetric vBMD; Tb.vBMD,trabecular vBMD; Tb.Meta.vBMD,external trabecular vBMD; Tb.Inn.vBMD, internal trabecular vBMD; Ct.vBMD,cortical vBMD; BV/TV,trabecular bone volume fraction; Tb.N,trabecular number; Tb.Th,trabecular thickness; Tb.Sp,trabecular separation; Tb.1/N.SD,trabecular bone heterogeneity; Ct.Th,cortical thickness; Ct.Po,intra-cortical porosity; Ct.Po.Dm,cortical pore diameter. \| \| \| \| \| \| \| \| \| \| \| \| \| \| |
| --- | --- | --- | --- | --- | --- | --- | --- | --- | --- | --- | --- | --- | --- | --- | --- | --- | --- | --- | --- | --- | --- | --- | --- | --- | --- | --- | --- | --- | --- | --- | --- | --- | --- | --- | --- | --- | --- | --- | --- | --- | --- | --- | --- | --- | --- | --- | --- | --- | --- | --- | --- | --- | --- | --- | --- | --- | --- | --- | --- | --- | --- | --- | --- | --- | --- | --- | --- | --- | --- | --- | --- | --- | --- | --- | --- | --- | --- | --- | --- | --- | --- | --- | --- | --- | --- | --- | --- | --- | --- | --- | --- | --- | --- | --- | --- | --- | --- | --- | --- | --- | --- | --- | --- | --- | --- | --- | --- | --- | --- | --- | --- | --- | --- | --- | --- | --- | --- | --- | --- | --- | --- | --- | --- | --- | --- | --- | --- | --- | --- | --- | --- | --- | --- | --- | --- | --- | --- | --- | --- | --- | --- | --- | --- | --- | --- | --- | --- | --- | --- | --- | --- | --- | --- | --- | --- | --- | --- | --- | --- | --- | --- | --- | --- | --- | --- | --- | --- | --- | --- | --- | --- | --- | --- | --- | --- | --- | --- | --- | --- | --- | --- | --- | --- | --- | --- | --- | --- | --- | --- | --- | --- | --- | --- | --- | --- | --- | --- | --- | --- | --- | --- | --- | --- | --- | --- | --- | --- | --- | --- | --- | --- | --- | --- | --- | --- | --- | --- | --- | --- | --- | --- | --- | --- | --- | --- | --- | --- | --- | --- | --- | --- | --- | --- | --- | --- | --- | --- | --- | --- | --- | --- | --- | --- | --- | --- | --- | --- | --- | --- | --- | --- | --- | --- | --- | --- | --- | --- | --- | --- | --- | --- | --- | --- | --- | --- | --- |
